# The Facioscapulohumeral muscular dystrophy region on 4qter and the homologous locus on 10qter evolved independently under different evolutionary pressure

**DOI:** 10.1186/1471-2350-8-8

**Published:** 2007-03-02

**Authors:** Monica Rossi, Enzo Ricci, Luca Colantoni, Giuliana Galluzzi, Roberto Frusciante, Pietro A Tonali, Luciano Felicetti

**Affiliations:** 1 Department of Neuroscience, Institute of Neurology, Catholic University of Sacred Heart, L.go A. Gemelli 8, 00168 Rome, Italy; 2 Center for Neuromuscular Diseases, UILDM, Via Prospero Santacroce, 5, 00167 Rome, Italy; 3 Fondazione Don Carlo Gnocchi, Via Maresciallo Caviglia, 30, 00194 Rome, Italy

## Abstract

**Background:**

The homologous 4q and 10q subtelomeric regions include two distinctive polymorphic arrays of 3.3 kb repeats, named D4Z4. An additional BlnI restriction site on the 10q-type sequence allows to distinguish the chromosomal origin of the repeats. Reduction in the number of D4Z4 repeats below a threshold of 10 at the 4q locus is tightly linked to Facioscapulohumeral Muscular Dystrophy (FSHD), while similar contractions at 10q locus, are not pathogenic. Sequence variations due to the presence of BlnI-sensitive repeats (10q-type) on chromosome 4 or viceversa of BlnI-resistant repeats (4q-type) on chromosome 10 are observed in both alleles.

**Results:**

We analysed DNA samples from 116 healthy subiects and 114 FSHD patients and determined the size distributions of polymorphic 4q and 10q alleles, the frequency and the D4Z4 repeat assortment of variant alleles, and finally the telomeric sequences both in standard and variant alleles.

We observed the same frequency and types of variant alleles in FSHD patients and controls, but we found marked differences between the repeat arrays of the 4q and 10q chromosomes. In particular we detected 10q alleles completely replaced by the 4q subtelomeric region, consisting in the whole set of 4q-type repeats and the distal telomeric markers. However the reciprocal event, 10q-type subtelomeric region on chromosome 4, was never observed. At 4q locus we always identified hybrid alleles containing a mixture of 4q and 10q-type repeats.

**Conclusion:**

The different size distribution and different structure of 10q variant alleles as compared with 4q suggests that these loci evolved in a different manner, since the 4q locus is linked to FSHD, while no inheritable disease is associated with mutations in 10qter genomic region. Hybrid alleles on chromosome 4 always retain a minimum number of 4q type repeats, as they are probably essential for maintaining the structural and functional properties of this subtelomeric region.

In addition we found: i) several instances of variant alleles that could be misinterpreted and interfere with a correct diagnosis of FSHD; ii) the presence of borderline alleles in the range of 30–40 kb that carried a qA type telomere and were not associated with the disease.

## Background

DNA repeats can be the source for an extraordinary amount of information about biological processes. As passive markers they provide clues for evolutionary events and processes of mutation and selection, as active agents repeats have remodeled the genome by causing ectopic rearrangements, by creating entirely new genes and by modifying and reshuffling existing genes. Specifically, segmental duplications involve the transfer of 1–200 kb blocks of genomic sequences to one or more locations in the genome and may contain various types of repeats [1]. These transfers have been shown to occur between homologous and non-homologous chromosomes [2,3]; their preference for pericentromeric and subtelomeric sites can be explained by a damage-control mechanism that facilitates insertion of chromosomal breakage products into poor-gene regions [4,5]. A particular case of interchromosomal duplication is that observed at the subtelomeric 4q35 and 10q26 loci [6]. The sequence homology between these subtelomeric regions is very high (nearly 99%) and is not restricted to a polymorphic array of 3.3 kb KpnI repeat units, designated D4Z4, but continues distally for at least 6 kb and proximally for nearly 42 kb [7,8]. The reduction in the number of 3.3 kb D4Z4 repeat to an array of 1–10 units at the 4q35 locus is tightly linked to FSHD, an autosomal-dominant myopathy with an estimated prevalence of 1/20.000 [9]. Similar contractions involving 10q26 D4Z4 repeats are not pathogenic. In normal subjects the single copy probe p13E-11 reveals polymorphic EcoRI fragments in the range 40 to 300 kb including the repeat array, whereas in sporadic and familial cases of FSHD the disease cosegregates with a EcoRI shorter fragment ranging between 10 and 40 kb [10-12]. The identification of a BlnI restriction site within each 10q repeat unit that is absent in the homologous 4q repeat [13] and the use of pulsed field gel electrophoresis (PFGE) allows to distinguish between the p13E-11 fragments of 4q and 10q origin, to study their number and size and to detect sequence exchanges between the two loci [14,15]. A high number of variant alleles has been detected in human population and is probably consistent with the occurrence of rearrangements between 4q and 10q homologous subtelomeric regions, even if the "de novo" translocation events were never detected. Sequence variations consist in the presence of BlnI-sensitive repeats (10q-type) on chromosome 4 or vice versa of BlnI-resistant repeats (4q-type) on chromosome 10. These exchanges were found in 20–30% of normal subjects and caused either a reduction of the number of BlnI-resistant fragments or an increase of the number of BlnI-resistant fragments in the genome [15], interfering with the correct assignment of DNA fragments to a specific chromosome pair and with an accurate genetic diagnosis of the FSHD [16]. Moreover in healthy individuals a biallelic variation was identified at the 4q region distal to D4Z4, named qA type and qB type. In FSHD patients the contracted D4Z4 fragments implicated in the disease are exclusively located on 4q alleles of qA type, while the 10q alleles always have a qA type telomere [17,18].

In this paper we performed a detailed analysis of 4q and 10q subtelomeric regions by characterizing the D4Z4 repeat assortment and the distal telomeric markers in Italian healthy subjects and FSHD patients, thus extending previous studies in other populations. We determined: i) the size distribution of polymorphic 4q alleles as compared with 10q alleles; ii) the frequency of variant 4q and 10q alleles; iii) the D4Z4 repeat assortment of 4q and 10q variant alleles; and finally; iv) the qA and qB telomeric sequences both in standard and variant alleles.

## Methods

Patients and controls Diagnostic criteria for FSHD followed the guidelines proposed by the European Expert Group on FMD [19]. Neurological examinations of 70 unrelated FSHD families for a total of 230 individuals (116 affected and 114 unaffected) were performed at Institute of Neurology of Catholic University of Rome and at the Centre for Neuromuscular diseases, U.I.L.D.M., Rome. For each FSHD family we chose only the proband, both for the study of size distribution of 4q–10q alleles and the types of variant arrays. Healthy subjects were chosen among hospital and laboratory staff as well among the spouses of FSHD families (genetically unrelated to FSHD patients) for a total of 106 individuals. DNAs were obtained from healthy subjects and patients after informed consent in agreement with the guidelines of the Ethical Committee of our Institution.

### Lymphocytes isolation from whole blood

Lymphocytes were isolated from peripheral blood sample, embedded in 0.5% Low Melt Agarose (Bio-Rad, Hercules, CA, USA) plugs and incubated with 0.5M EDTA, 1% Sarcosyl (Sigma, St Louis, MO, USA) and 1 mg/ml Proteinase K for 24–48 hrs at 50°C (Roche, Mannheim, Germany), according to the procedure described by Pharmacia.

### Restriction endonuclease digestion of DNA in agarose blocks and PFGE

1/4 of plug, corresponding to 0.5 × 10^6 ^cells, was digested with the following restriction enzymes: EcoRI (Roche, Mannheim, Germany), BlnI (Amersham Pharmacia Biotech, Buckingamshire, U.K.) and XapI (Fermentas). Single (EcoRI, XapI) and double digestions (EcoRI/BlnI) were performed with 60/150 U of enzyme added in three equal portions, at 1h intervals and incubated overnight.

PFGE was performed at 10°C in a Pulsaphor Electrophoresis unit with HEX electrode (Pharmacia LKB). High Strength Analytical Grade Agarose (Bio-Rad, Hercules, CA, USA) was used to prepare 1.2% gel in 0.5 tris-borate-EDTA. The running procedure was performed at 300 V in 4 cycles: the first cycle was 1 h 30' with pulse of 0.3 s; the second was 3 h 30' with pulse of 0.5 s, the third was 4 h with pulse of 1 s and the fourth was 2 h 30' with pulse of 5 s [20].

### Southern Blot analysis

After electrophoresis the DNA was transferred to Nylon membrane Hybond N+ (Amersham Pharmacia Biotech, Buckingamshire, U.K.) for hybridization. The probes were labelled by random priming with [α-^32^P]ATP using the Multiprime DNA labeling system (Amersham Pharmacia Biotech, Little Chalfont, UK). Both the single copy p13E-11 probe and the KpnI repeat sequence were obtained from 4.9 kb KpnI fragment subcloned in pUC21 [21]. For the KpnI probe we used the 1.179 bp fragment derived from BamHI digestion of the 3.3 kb KpnI repeat. Hybridizations were performed overnight at 42°C in a hybridization mixture containing 50% deionized Formamide, 3 × SSC, 5 × Denhardt's, 200 μg/ml Salmon Sperm and 10% Dextran Sulfate. The membranes were washed in 2 × SSC at room temperature and then in 0.5 × SSC/0.1%SDS at 58°C. Labelling and hybridization with D4S139 of digested genomic DNA were performed as previously described [21].

### qA and qB telomeric markers

**Figure 1:**
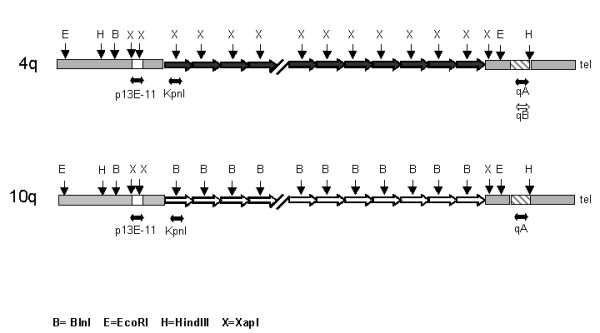
**4q and 10q subtelomeric region**. Positions of DNA probes and restriction enzymes utilized in the study are indicated.

To perform the allele-typing for qA and qB, the DNA of healthy and affected subjects was digested with HindIII restriction enzyme (Roche Mannheim, Germany) (Fig. 1). The fragments were separated with PFGE, transferred to a Nylon membrane and hybridized with probes qA and qB, supplied by S. M. van der Maarel [17]. The hybridizations were performed overnight at 60°C in a mixture containing 0.125 M Na_2_HPO_4 _(pH 7.2), 0.25 M NaCl, 1 mM EDTA and 7% SDS. The membranes were washed in 2 × SSC at 42°C and then in 0.1 × SSC and 0.1% SDS at 65°C for 15 minutes.

### Statistical analysis

The mean values of 4q and 10q allele sizes derived from healthy and FSHD subjects were compared using Mann-Whitney U-test and T-student test. The size distribution of 4q and 10q polymorphic fragments showed a bimodal distributions when divided into 20 kb classes. Upon visual inspection of the distribution we decided to partition each of them in 2 clusters. Cluster centers and number of chromosomes falling in each cluster were obtained with the program Quickcluster as implemented in SPSS v. 6.0.

## Results

### Canonical and non canonical pattern of 4q and 10q alleles in human subjects

We defined an individual as carrying a canonical pattern of alleles when, after PFGE analysis and hybridization with p13E-11 probe, we observed four restriction fragments with EcoRI and only two restriction fragments after double digestion EcoRI/BlnI: the alleles of 10q origin containing BlnI sensitive repeats were converted into 2.8 kb fragments and run out of the gel, while the alleles of 4q origin containing BlnI-resistant repeats were preserved and reduced by 3 kb due to a single BlnI restriction site 3 kb distal to the proximal EcoRI restriction site (Fig. 1). We defined an individual as carrying a non canonical pattern of alleles when the number of BlnI-resistant fragments are different from two (nullisomy, monosomy, trisomy, tetrasomy) [15,20].

### Size distribution of 4q and 10q alleles

#### Healthy individuals (Fig. 2a)

**Figure 2:**
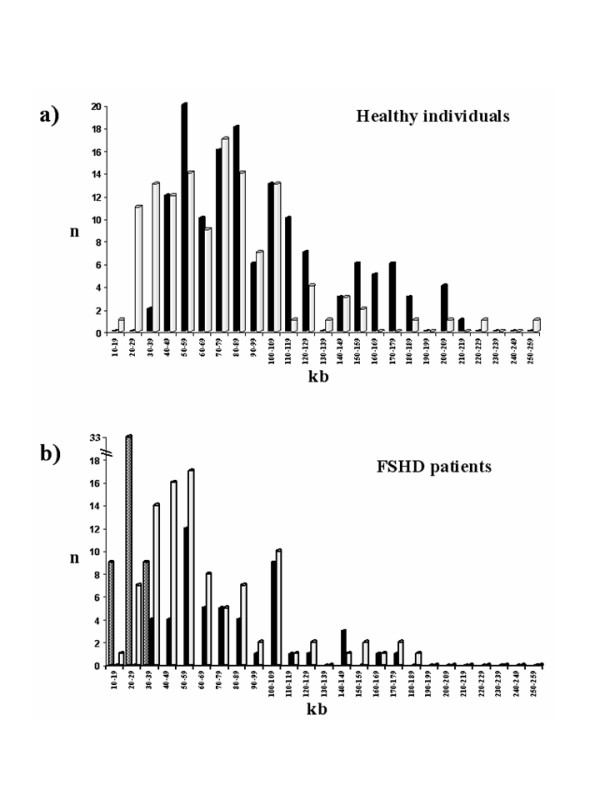
**Size distribution of 4q and 10q alleles in healthy and FSHD affected subjects**. The size distributions of 4q and 10q alleles detected in healthy subjects (panel a) and FSHD patients (panel b) are represented. The size of EcoRI fragments is expressed in kb, with 10 kb intervals. Black bars: 4q wild-type alleles; grey bars: disease-associated 4q alleles; white bars: 10q alleles.

Only 67% of 106 healthy subjects showed, after p13E-11 hybridization, a canonical pattern of alleles (Tab. 1). In these individuals the size of 4q alleles ranged between 30 and 200 kb (mean 90.68 ± 42.84) and displayed a bimodal distribution, with a larger peak at mean values of 80 kb and a smaller peak at 160 kb. The size of 10q alleles ranged between 10 and 250 kb (mean 74.36 ± 41.88). The difference in size distribution of 4q and 10q alleles was significant (p = 0.002 by T Student test and p = 0.0006 by Mann-Whitney test), as previously reported by Wijmenga et al. [22].

**Table 1:** Canonical and non canonical patterns in healthy subjects and FSHD patients

	**Healthy **	**FSHD**
- **Canonical pattern**	71 (**67%**)	51 (**73%**)
- **Non canonical pattern**	35 (**33%**)	19 (**27%**)
*Nullisomy*	0	1
*Monosomy*	16	5
*Trisomy*	10	12
*Tetrasomy*	0	0
*Complex rearrangements*	7	1
*p13E-11 deletions*	2	0

**Total number**	106	70

#### FSHD familial cases (Fig. 2b)

Among 70 FSHD probands, 73% showed a canonical patterns. The mean value of the sizes of the 4q alleles not implicated in the disease was 76.73 ± 33.90 kb, significantly lower than that found in the controls carrying a pair of wild-type 4q alleles (p = 0.037 by T Student test and p = 0.042 by Mann-Whitney test). This result was in contrast with the even distribution of the size of 10q alleles in both healthy and affected subjects. The mean value of the size of the disease- related allele was 25.12 ± 5.37 kb.

#### qA and qB Telomeric markers

A biallelic variation, named qA and qB type, was identified at 4q telomeric region distal to D4Z4 [17]. We analysed the telomeric sequences (qA/qB allele-typing) of 66 healthy and 40 FSHD individuals of our groups (see Methods) and reported the results in table 3. In FSHD patients the small 4q alleles linked to the disease were exclusively of A type, in agreement with the results obtained in the Dutch population [17]. Since qA alleles were reported to be on the average longer than qB alleles [23], the overrepresentation of qA telomeres in our control population could explain the higher mean value of 4q allele sizes in healthy subjects compare to FSHD patients (Tab. 2).

**Table 2:** Typing of qA and qB telomeres of 4q alleles from healthy subjects and FSHD patients

**Healthy subjects**		**FSHD patients (normal allele)**		**FSHD patients (short allele)**	
4qA	4qB	4qA	4qB	4qA	4qB

45 (68%)	21 (32%)	23 (57%)	17 (43%)	40 (100%)	0 (0%)

TOT: 66 alleles		TOT: 40 alleles		TOT: 40 alleles	

**Table 3:** Number and type of variant alleles in healthy subjects and FSHD patients

	**Chr. 4 alleles**		**Chr. 10 alleles**	
	**Healthy**	**FSHD**	**Healthy **	**FSHD**
Total number of alleles	212	140	212	140

**Variant alleles (total)**	19(9%)	8(6%)*	13(6%)	13(9%)
homogeneous KpnI array	0	0	12	11
heterogeneous KpnI array	19	8	1	2
**p13E-11 deletion **	2	0	0	0

*only one variant 4q allele is disease-related

**Figure 3:**
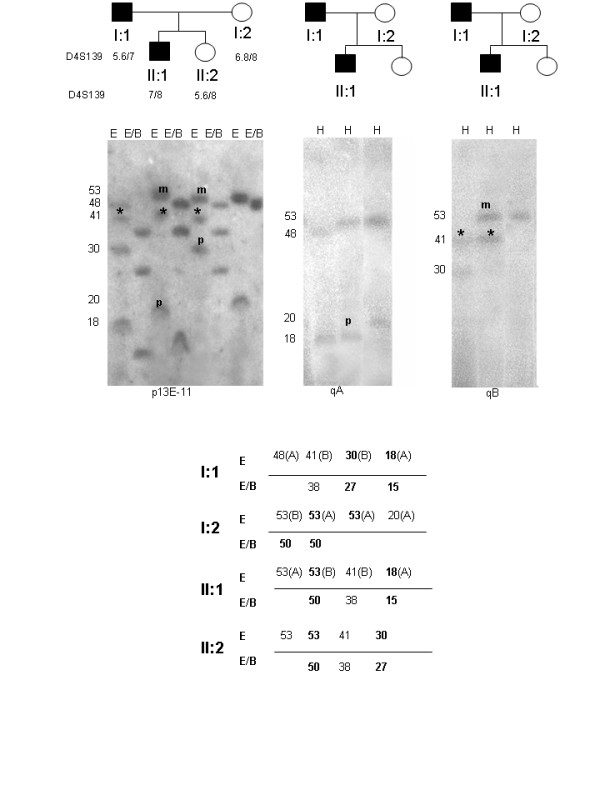
**4q and 10q allelic pattern of a FSHD family after hybridization with p13E-11 and qA-qB telomeric markers**. a) DNA samples were digested with EcoRI (E) and EcoRI/BlnI (E/B) enzymes, separated with PFGE and hybridized with p13E-11 probe. The proband (I:1) shows a trisomic pattern with two 4q alleles of 18 and 30 kb and two 10q alleles of 41 (BlnI-resistant) (*) and 48 kb (BlnI-sensitive). The affected son (II:1) is also trisomic and inherited from the father the 4q FSHD allele of 18 kb and the10q of 41 kb (*) and from the mother the 4q of 53 kb and a similar size10q allele. The unaffected daughter inherited from the father the 4q allele of 30 kb and the 10q BlnI-resistant allele of 41 kb (*) and from the mother the 4q and 10q fragments of 53 kb. The segregation data for the probe D4S139 showed that II:1 and II:2 inherited the same 4q allele from the mother (I:2), but different 4q alleles from the father (I:1). This suggest that the BlnI-resistant allele of 41 kb is a 10q variant; b) DNA samples were digested with HindIII (H), separated with PFGE and subsequently hybridized with qA and qB probes: the short 4q allele (18 kb) is qA type, while the 4q allele of 30 kb is qB type. All the standard 10q alleles of 48, 53 and 20 kb have qA type telomeres, while the variant 10q allele of 41 kb is qB type. This variant 10q allele carries the Bln1-resistant repeat array and the distal telomeric sequence of 4q-type. The 10q origin of this allele is confirmed by absence of segregation with D4S139 probe. The DNA sample of the subject II:2 was not sufficient for qA/qB Southern Blot analysis and it was not included in figure 3. In the diagram below the alleles observed after EcoRI and EcoRI/BlnI digestion are shown: the 4q alleles are in bold and the telomeres (A or B) are in brackets.

Among healthy subjects we observed two 4q alleles of 33 and 38 kb (Fig. 2a). They showed telomeric sequences of qA type. Furthermore we described four affected individuals carrying two 4q alleles shorter than 40 kb : in each case one 4q allele was FSHD-linked, the other was also present in healthy members of the family. Among the 4q short alleles not linked to the disease two of them of 30 and 37 kb had qB type telomeres (Fig. 3), the other two of 33 and 38 kb had qA type telomeres. The proband carrying the allele of 33 kb also had a FSHD-linked 4q of 29 kb (qA) and the proband carrying the allele of 38 kb had a FSHD-linked 4q of 26 kb (qA). The phenotype of both probands were compatible to the length of their FSHD-linked 4q [12]. We did not observe relevant differences between the clinical phenotype of these patients carrying both 4qA alleles shorter than 40 kb and the other affected members of the families. Since the phenotypic expression of FSHD is extremely variable both within and between families, our two cases are not sufficient to confirm or reject the theory of dosage effect in FSHD patients [24].

### 4q and 10q allelic patterns in healthy subjects and FSHD patients

In healthy individuals we found a change in the disomic pattern of alleles in 35 cases (33%): 10 (9%) were trisomic, 16 (15%) monosomic and 7 (7%) displayed more complex rearrangements (Tab. 1). Finally, in 2 individuals, we found only three EcoRI restriction fragments, suggesting a deletion that involves p13E-11 sequences. In the first individual we did not find any residual D4Z4 repeats on 4q, in agreement with the observation that cytogenetic deletions encompassing p13E-11 and the whole set of D4Z4 repeats are not pathogenic. It is generally accepted that at least one KpnI repeat is required for the development of the disease [25,26]. In the other case hybridization with KpnI probe detected a BlnI resistant fragment of 120 kb, corresponding to a residual array of 35 D4Z4 repeats. The presence of a number of D4Z4 above the threshold number of 10 does not lead to the disease despite the deletion of p13E-11 sequence.

**Figure 4:**
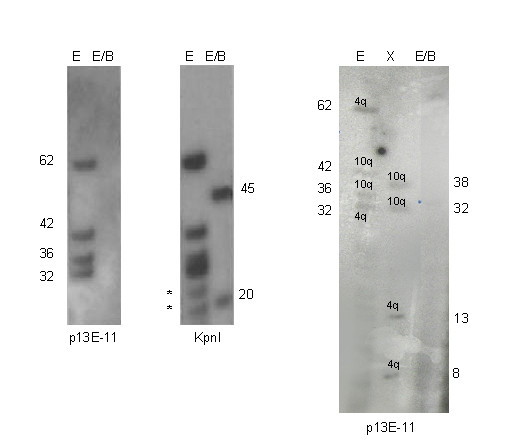
**Nullisomic pattern**. The patient shows four EcoRI bands after p13E-11 hybridization and none after double digestion with EcoRI/BlnI (nulisomic pattern). After hybridization with KpnI probe two BlnI resistant band are visible (45 and 20 kb): we can not guess the chromosomal origin of these alleles. To resolve the structure of the alleles we digested the sample with XapI enzyme which cuts the 4q-type repeats only (see Methods). After hybridization with p13E-11 we observed 4 XapI-resistant bands (38, 32, 13 and 8 kb): the 38 and 32 kb bands correspond to the EcoRI fragments of 42 and 36 kb fragments (XapI fragments are 4 kb smaller than EcoRI fragments). On the contrary the 13 and 8 kb fragments are stretches of the hybrid 4q alleles: the 62 kb EcoRI allele is composed by a BlnI-sensitive fragment (XapI-resistant) of 13 kb and a BlnI-resistant fragment of 45 kb (13+45 = 58 kb). The 32 kb EcoRI allele is composed by a BlnI-sensitive fragment (XapI-resistant) of 8 kb and a BlnI-resistant fragment of 20 kb (8+20 = 28 kb). The 4q variant alleles are represented in figure 5 (n.15 and 16). The FSHD 4q allele of 32 kb segregated also in other affected members of the family and its origin was confirmed by D4S139 probe (data not shown). * aspecific bands.

Among the 70 FSHD probands we found that 73% carried a canonical pattern of alleles (Tab. 1), the remaining subjects (27%) displayed different kinds of variant alleles giving origin to a pattern of trisomy in 12 patients (17%), monosomy in 5 (7%) and nullisomy in one of them (Fig. 4).

### Analysis of structural organization of variant 4q and 10q alleles in healthy and affected subjects

After hybridization with p13E-11, the filters were stripped and re-hybridized with the multicopy KpnI probe (see Methods). If the 4q and 10q alleles were homogeneous in D4Z4 repeat constitution, KpnI hybridization confirmed the canonical pattern observed with p13E-11. On the contrary, if the 4q and 10q alleles had a non homogeneous repeat array, additional bands were observed after hybridization with KpnI (Fig. 4). The bands are stretches of BlnI-resistant repeats separated from p13E-11 sequence by BlnI-sensitive repeats. We assigned the fragments to their original alleles through the study of the segregation with p13E-11 and D4S139 (4q specific) probes in the family. After that we reconstructed the repeat array assortment of the variant allele by subtracting the size of the BlnI-resistant band (observed with KpnI probe) from the length of the EcoRI band (observed with p13E-11 probe).

In more complex cases and where we could not study the allelic segregation *in *the family, we performed an additional Southern blot analysis with the DNA samples digested with the restriction enzyme XapI (Fig. 4). This enzyme shows the opposite characteristics to BlnI: XapI restriction sites are present in the 4q-type repeats and are absent in the 10q-type [27].

In conclusion we named as "standards" those allele in which both the 4q and 10q alleles carried a homogeneous, uninterrupted array of BlnI-resistant (4q-type) or BlnI-sensitive (10q-type) repeats, respectively (Fig. 1). We defined as "variants" those 4q and 10q alleles with different assortment of D4Z4 repeats. Among variant alleles we detected hybrid types (alleles with a mixture of 4q-type and 10q type repeats) and homogeneous types.

In healthy subjects we analysed a total number of 212 alleles of either 4q or 10q types and identified 19 4q variant alleles (9%) and 13 10q variant alleles (6%) (Tab. 3).

A similar frequency was observed in the affected individuals: among the 140 alleles analysed, 8 variant 4q (6%), and 13 variant 10q (9%) alleles were encountered (Tab. 3).

**Figure 5:**
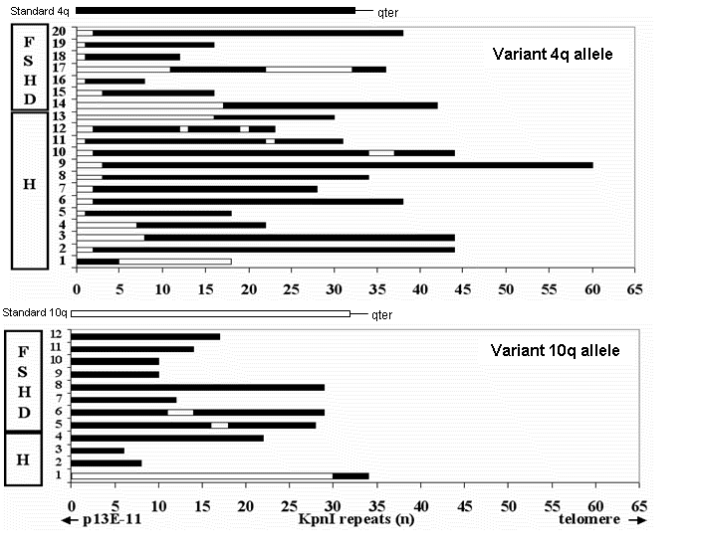
**Analysis of structural organization of variant 4q and 10q alleles in healthy subjects and FSHD patients**. White bars: BlnI-sensitive repeats; Black bars: BlnI-resistant repeats. Variant 4q alleles: N° 1–13 refer to healthy subjects (H), N° 14–20 to FSHD patients. Variant 10q alleles: N° 1–4 refer to healthy subjects (H), N° 5–12 to FSHD patients.

In figure 5 we described the 4q and 10q variant alleles in which we could reconstruct the D4Z4 repeat assortment. Since family study or additional DNA samples for XapI digestions were not always available, we were not able to reconstruct the D4Z4 repeat constitution of the variant alleles in all individuals. However we observed that even the 4q alleles not included in the diagram showed a stretch of BlnI-sensitive repeats followed by BlnI-resistant repeats, while all the 10q variant alleles carried an uninterrupted array of BlnI-resistant repeats.

#### 4q variant alleles (10q repeats on chromosome 4q)

We observed BlnI-sensitive repeats upstream the BlnI-resistant units on 4q chromosome in 26 out of 27 variant alleles (Fig. 5). Only one allele from a healthy subject showed 10q-type repeats into the terminal part of a 4q array: the 4q origin of the allele was confirmed by the segregation analysis using D4S139 probe. It is worth noting that in all cases the variant 4q alleles had a heterogeneous mixture of BlnI-resistant (4q-type) and BlnI-sensitive (10q-type) D4Z4 repeat units (Tab. 3).

Among FSHD patients we found one case in which the disease allele was hybrid and carried BlnI-sensitive repeats mimicking a 10q allele (allele n.16 Fig. 5).

The size of the variant 4q alleles ranged between 40 and 200 kb (corresponding to 10 and 60 repeat units) and there was no preferential involvement of a specific class of alleles. In 14 cases a small number of 10q-type repeats were present on 4q alleles (from 1 to 3 repeats). In 4 alleles (10-11-12-17) we found evidence of multiple clusters of BlnI-sensitive repeats interspersed within BlnI-resistant units.

#### 10q variant alleles (4q repeats on chromosome 10q)

In 25 out of 26 cases (96%) BlnI-resistant D4Z4 repeats were detected on the 10q chromosome at the proximal level, close to the p13E-11 site (Fig. 5). In contrast to the heterogeneity of the 4q variant alleles, the 10q variant alleles were homogeneous in 23 cases (88%) (Tab. 3). The set of 4q-type repeats on 10q chromosome was uninterrupted, only in 2 cases few BlnI-sensitive repeats were interspersed within BlnI-resistant arrays. Only in one case the 4q-type repeats were found in the terminal part of the 10q repeat array. The size of variant 10q alleles varied between 20 and 120 kb and the number of 4q-type repeats (ranging between 4 and 29) was higher than the number of 10q-type repeats observed on 4q variant alleles (ranging between 1 and 17).

#### Telomeric markers

**Figure 6:**
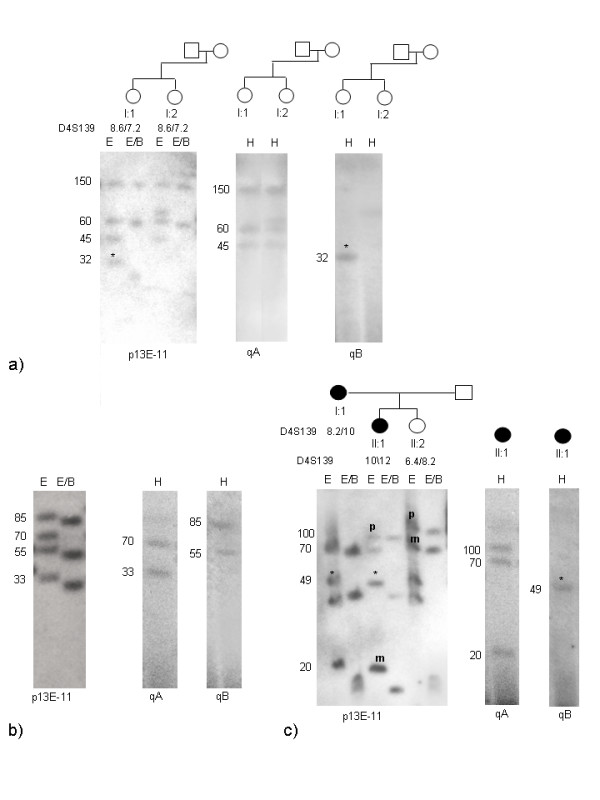
**10q alleles with qB telomere**. Allelic pattern of individuals carrying 10q alleles with a qB type telomere. a) The segregation study with the D4S139 probe demonstrates that the two sisters inherited the same 4q alleles of 150 and 60 kb. I:1 shows a trisomic pattern with a BlnI-resistant 10q of 32 kb (*) and a standard 10q of 45 kb. The analysis of the telomeric sequences reveals that the variant 10q allele of 32 kb has a qB telomere. The two 4q alleles and the standard 10q allele of 45 kb have qA telomeres. b) The patient is trisomic and carries a FSHD allele of 33 kb and a standard 10 q allele of 70 kb with a qA telomere. We can not distinguish the 10q variant allele from the 4q standard alleles. Since both the 85 kb and 55 kb show a qB telomere, we deduce that the variant 10q allele carries a qB telomere. c) The 4q short allele of 20 kb segregates with FSHD in the family. The affected daughter (II:1) is trisomic and inherited from the mother (I:1) both the FSHD-linked 4q of 20 kb and the BlnI-resistant 10q allele of 49 kb (*) and from the father a standard 10q allele of 70 kb and a 4q standard allele of 100 kb. The analysis of the telomeric sequences shows that the variant 10q allele of 49 kb has a qB telomere, while the 10q standard allele of 70 kb and both 4q alleles of 100 and 70 kb have a qA telomere. The 10q variant allele of 49 kb is represented in figure 5 (n.11).

We were interested to extend the study of qA and qB telomeric sequences to 4q and 10q variant alleles. We analysed twelve 4q variant alleles: nine of them showed an A type telomere, three a B type. Among the twelve 10q variant alleles studied carrying a homogeneous set of BlnI-resistant repeats, eight were of A type and four of B type (Fig. 3 and Fig. 6), while standard 10q alleles always displayed A type telomeres.

## Conclusion

The high level of sequence homology between the 4q and 10q loci would imply for both polymorphic regions a similar size distribution and recombination products. This is not the case: 10q alleles displayed a different size distribution as compared with 4q alleles with a high percentage of alleles shorter than 40 kb (18%). The differences are more evident when we analysed the structure of variant alleles. 4q variant alleles were heterogeneous and always contained a mixture of BlnI-sensitive (10q-type) and BlnI-resistant (4q-type) repeats, on the contrary 10q variant alleles usually displayed a homogeneous set of BlnI-resistant (4q-type) repeats. The variant alleles revealed that such interactions probably occurred in the past, but the different structures of 4q and 10q variant alleles support the idea that the exchanges between the two loci are rare and these subtelomeric regions appear to evolve independently under different evolutionary pressure. Since reduction in the number of D4Z4 on 4q locus results in FSHD, it is not surprising that 4q chromosomes have incurred a strong selection pressure during human evolution. Gabellini et al. reported that a repressive protein complex binds a specific sequence within each D4Z4 repeat unit on chromosome 4. The reduction in the number of repeats in FSHD patients abolishes this binding and results in the loosening of the transcriptional repression at 4q region and in the overexpression of upstream genes (FRG2, FRG1, ANT1) [28,29]. Accordingly we observed that 4q type repeats are preserved on 4q chromosome as they are probably essential for maintaining the structural and functional properties of this subtelomeric region. In particular the distal repeats, close to qA and qB sequences, were always maintained on chromosome 4.

Moreover we described qB telomeres on 10q variant alleles carrying homogeneous 4q-type repeat arrays. This suggests that qB sequences are not exclusively associated with 4q chromosomes, but they are always linked to BlnI-resistant repeats.

It was proposed that an ancestral duplication event generated 4q and 4p telomeric regions, then 4q sequences diverged and gave origin to a 4qA type telomere that was duplicated onto chromosome 10. A more recent duplication of telomeric sequences from 4p onto 4q gave origin to 4qB-type telomere [7]. Therefore the presence of qB on chromosome 10 demonstrates that a telomeric sequence exchange has occurred between the 4qB and 10q chromosomes after the duplication events.

We reported a detailed molecular analysis of the 10q and 4q subtelomeric regions in FSHD patients and controls with the aim of characterizing the D4Z4 repeat array assortment and the distal telomeric region (qA and qB) of 4q and 10q alleles. We obtained new interesting findings about the structure of these subtelomeric regions that should be taken into account for a correct molecular diagnosis and genetic counseling of FSHD. In some cases the chromosome origin of the p13E-11 fragments could be misinterpreted: i) we found several 10q variant alleles with a BlnI-resistant array and a size smaller than 40 kb (Fig. 5) mimicking a FSHD-linked alleles; ii) in one patient we observed a 4q FSHD-linked allele carrying BlnI-sensitive repeats upstream the BlnI-resistant repeats, mimicking a 10q allele (Fig. 4).

Furthermore we studied in a different population the 4qter polymorphism distal to D4Z4 described in a previous Dutch study [17] and confirmed the FSHD association only with qA type sequence, but we also detected the presence of borderline alleles in the range of 30–40 kb of qA type, not associated with the disease. This suggests that other concomitant factors, either genetic or environmental, are implicated in the pathogenesis of the FSHD. It appears that the 4qA allele-typing is necessary but not sufficient to assign the pathogenic role to a 4q short allele.

## Abbreviations

Facioscapulohumeral Muscular Dystrophy: FSHD

## Competing interests

The author(s) declare that they have no competing interests.

## Authors' contributions

MR performed laboratory work, results collection and wrote the paper. ER, GG and LF designed and coordinate the study. PAT recruited subjects and participated in clinical diagnosis, LC performed laboratory work and results collection. RF recruited subjects. LF supervised the study and wrote the paper.

All authors read and approved the final manuscript.

## Pre-publication history

The pre-publication history for this paper can be accessed here:


